# EPINTLM: enhancer–promoter prediction with pretrained k-mer embeddings and residual cross-attention

**DOI:** 10.1093/bib/bbag064

**Published:** 2026-02-16

**Authors:** Thi Lan Nguyen, Hien Quang Kha, Phat Ky Nguyen, Minh Huu Nhat Le, Duc-Trong Le, Nguyen Quoc Khanh Le

**Affiliations:** VNU University of Engineering and Technology, Vietnam National University, 144 Xuan Thuy, Cau Giay, Hanoi 100000, Vietnam; AIBioMed Research Group, Taipei Medical University, 250 Wuxing Street, Taipei 110, Taiwan; AIBioMed Research Group, Taipei Medical University, 250 Wuxing Street, Taipei 110, Taiwan; International Master/Ph.D. Program in Medicine, College of Medicine, Taipei Medical University, 250 Wuxing Street, Taipei 110, Taiwan; AIBioMed Research Group, Taipei Medical University, 250 Wuxing Street, Taipei 110, Taiwan; International Master/Ph.D. Program in Medicine, College of Medicine, Taipei Medical University, 250 Wuxing Street, Taipei 110, Taiwan; AIBioMed Research Group, Taipei Medical University, 250 Wuxing Street, Taipei 110, Taiwan; International Master/Ph.D. Program in Medicine, College of Medicine, Taipei Medical University, 250 Wuxing Street, Taipei 110, Taiwan; VNU University of Engineering and Technology, Vietnam National University, 144 Xuan Thuy, Cau Giay, Hanoi 100000, Vietnam; AIBioMed Research Group, Taipei Medical University, 250 Wuxing Street, Taipei 110, Taiwan; In-Service Master Program in Artificial Intelligence in Medicine, College of Medicine, Taipei Medical University, 250 Wuxing Street, Taipei 110, Taiwan; Translational Imaging Research Center, Taipei Medical University Hospital, 252 Wuxing Street, Taipei 110, Taiwan

**Keywords:** enhancer–promoter interaction, genomic sequence, multimodal feature, cross-attention mechanism, residual connection, pretrained large language models

## Abstract

Enhancer–promoter interactions (EPIs) play an important role in gene regulation, yet experimental mapping remains costly and limited in coverage. As a result, computational approaches are commonly evaluated under curated benchmark datasets, which pose challenges related to long-range sequence modeling, multimodal feature integration, and reproducible preprocessing. In this study, we present EPINTLM (Enhancer–Promoter Interaction Nucleotide Transformer Large Model), a deep learning framework designed to investigate architectural strategies for EPI prediction under standardized benchmark settings. EPINTLM integrates DNA sequence representations and genomic features by leveraging pretrained k-mer embeddings from the Nucleotide Transformer and explicitly modeling intra- and inter-sequence dependencies through residual self-attention and bidirectional cross-attention. We additionally introduce a unified preprocessing pipeline to improve training efficiency and reproducibility, and perform post hoc motif analysis to provide limited interpretability of learned sequence patterns. Evaluated on a widely used benchmark across six human cell lines, EPINTLM achieves competitive area under the receiver operating characteristic curve (AUROC) and area under the precision-recall curve (AUPR) performance relative to existing methods, with ablation studies highlighting the contributions of cross-attention and residual aggregation. These results demonstrate the utility of explicit cross-attention designs for paired regulatory sequence modeling within current benchmark constraints.

## Introduction

Predicting enhancer–promoter interactions (EPIs) is a critical problem in computational genomics, with broad implications for understanding gene regulation, disease mechanisms, and cell-type-specific transcriptional control [1]. While experimental techniques like Hi-C and ChIA-PET provide gold-standard evidence, they are expensive and not scalable across tissues [2], motivating the need for efficient computational alternatives. Recent advances in machine learning have shown that EPIs can be inferred from genomic and epigenomic data, using models that incorporate chromatin features, such as histone marks and transcription factor binding [3]. Early approaches relied on hand-crafted features and shallow classifiers, while deep learning models have demonstrated improved performance through automatic feature extraction from raw DNA sequences [2]. However, EPI prediction presents distinct challenges for machine learning: (i) modeling long-range dependencies between enhancer and promoter regions that may be tens to hundreds of kilobases apart; (ii) learning generalizable representations across diverse cellular contexts; and (iii) effectively integrating multimodal biological signals. Existing attention-based models, such as TransEPI [4], EPIVAN [2] partially address these problems by applying attention mechanisms within concatenated enhancer–promoter sequences. Yet, they often require large training datasets and only focus on intra-sequence feature learning through self-attention [5], lacking direct bidirectional interaction modeling between enhancers and promoters via cross-attention. To address these limitations, we propose **EPINTLM** (Enhancer–Promoter Interaction Nucleotide Transformer Large Model), a novel deep learning architecture that explicitly models bidirectional enhancer–promoter regulatory interactions. EPINTLM integrates pretrained DNA language model embeddings with tailored attention mechanisms, leveraging k-mer representations from the Nucleotide Transformer. By incorporating inter-sequence cross-attention, EPINTLM directly captures regulatory dependencies between enhancers and promoters, while residual connections help stabilize information flow across deep layers. In addition, our architecture fuses sequence and epigenomic features through a modular attention framework, enabling both accuracy and interpretability.

We evaluate EPINTLM under a widely used benchmark setting across six human cell lines, following established protocols for computational EPI prediction. Within this controlled evaluation framework, EPINTLM achieves competitive AUROC and AUPR performance relative to existing methods. Ablation studies are used to assess the impact of architectural components, demonstrating the roles of explicit cross-attention and residual aggregation in stabilizing learning and balancing discrimination performance. These experiments are intended to examine modeling choices under standardized benchmark assumptions, rather than to provide genome-wide or context-complete inference of enhancer–promoter regulation.

In summary, the main contributions of this work are:

- A dual-attention deep learning architecture designed to explicitly model intra- and inter-sequence relationships between enhancer and promoter regions under benchmark evaluation settings.
- An analysis of bidirectional cross-attention as an architectural mechanism for paired regulatory sequence modeling.
- The integration of pretrained k-mer embeddings from the Nucleotide Transformer to support contextual sequence representation learning.
- A unified and reproducible preprocessing pipeline for combining genomic and sequence features.
- A systematic benchmark-based evaluation against five representative state-of-the-art methods.

## Related work

### Pretrained DNA embeddings

 Early sequence-based EPI prediction models such as SPEID [6] employed convolutional networks with one-hot encoded inputs, limiting their ability to capture contextual or semantic relationships between enhancer and promoter regions. Subsequent models like EPIVAN [2] incorporated pretrained DNA embeddings to enhance representation learning; however, these were static k-mer vectors (e.g. dna2vec [7]) that lacked deep contextualization. More recently, EPIPDLF [8] adopted embeddings derived from DNABERT, allowing some degree of fine-tuning during training. Despite this improvement, these embeddings were extracted only from the input layer and bypassed intermediate transformer layers. In contrast, our approach leverages the full representational depth of the Nucleotide Transformer [9]. We pass tokenized enhancer and promoter sequences through the entire model and perform mean pooling on the final hidden layer, yielding embeddings that capture both local sequence motifs and long-range dependencies. Importantly, these contextualized embeddings remain trainable throughout downstream learning, allowing task-specific adaptation while retaining the expressive power of the foundation model.

### Attention mechanisms and residual learning

Several prior works have explored attention in EPI prediction. EPIANN [10] introduced sequence-level pairwise attention to align enhancer and promoter regions, while EPIVAN [2] employed attention as a postconcatenation weighting mechanism. EPIPDLF [8] improved intra-sequence modeling through independent multi-head self-attention for enhancers and promoters. However, to our knowledge, no previous study has employed explicit *cross-attention* to model bidirectional EPIs, where one sequence attends to another (e.g. enhancer as query, promoter as key/value, and vice versa). EPINTLM introduces this cross-attentional mechanism to directly capture inter-sequence regulatory dependencies, which we show to be particularly effective on transcriptionally complex cell types like HeLa. In addition, we integrate residual connections across attention and recurrent layers to ensure information preservation. These connections contribute to improved AUROC across cell lines by enhancing overall classification robustness, not just precision on positive samples.

### Data processing and training efficiency

The preprocessing pipeline also plays a crucial role in model efficiency and reproducibility. EPIPDLF [8] constructs training datasets by dynamically combining separate sequence and genomic feature loaders during runtime. While flexible, this design introduces complexity in data access and management. In contrast, we propose a unified preprocessing strategy that encodes labels, genomic signals, and tokenized sequences into a single serialized dataset format, SeqGenDataset. This compact representation is stored as a PyTorch .pt file, enabling fast, index-based retrieval of all input modalities and significantly reducing training overhead.

## Proposed method

### Overview

We formulate EPI prediction as a binary classification task. Given an enhancer sequence $E$, a promoter sequence $P$, and associated genomic features $G$, the goal is to learn a function $f: (E, P, G) \rightarrow \{0, 1\}$ that outputs the probability of a functional interaction between $E$ and $P$. A prediction of 1 indicates an interacting pair, while 0 denotes non-interaction. Model performance is primarily evaluated using AUROC and AUPR, both of which are standard for imbalanced classification problems.

### Dataset preparation

We adopt the *TargetFinder* dataset [1] to enable fair comparison with existing EPI predictors. This dataset comprises labeled enhancer–promoter pairs derived from high-resolution Hi-C data across six human cell lines: **GM12878** (lymphoblastoid), **HeLa-S3** (cervical carcinoma), **HUVEC** (umbilical vein endothelial), **IMR90** (fetal lung fibroblast), **K562** (leukemia-derived mesodermal), and **NHEK** (epidermal keratinocyte). A defining challenge of this dataset is its severe class imbalance: fewer than 5% of enhancer–promoter pairs are labeled as interacting, while the remaining 95% are negatives. This sparsity mirrors biological reality but complicates effective model training and evaluation. Sample statistics for each cell line are summarized in Table 1.

**Table 1:** Dataset statistics for the six human cell lines used in the benchmark evaluation

**Cell line**	**Original Pos**	**Original Neg**	**Train Pos**	**Train Neg**	**Test Pos**	**Test Neg**
GM12878	2113	42 200	38 040	37 980	211	4220
HeLa-S3	1740	34 800	31 320	31 320	174	3480
HUVEC	1524	30 400	27 440	27 360	152	3040
IMR90	1254	25 000	22 580	22 500	125	2500
K562	1977	39 500	35 600	35 550	197	3950
NHEK	1291	25 600	23 240	23 040	129	2560

“Pos” denotes positive EPIs and “Neg” denotes negative samples.

To mitigate this imbalance and follow established protocols (e.g. *EPIANN* [10]), we employ a multi-stage data preprocessing and augmentation pipeline:

1. We begin with an imbalanced dataset $D$, where positive samples are heavily outnumbered.
2.
$D$ is split into a training set $D_{\mathrm{train}}$ (90%) and a test set $D_{\mathrm{test}}$ (10%), using stratified sampling to preserve class ratios.
3. To counter class imbalance in $D_{\mathrm{train}}$, we perform data augmentation to generate a balanced dataset $D_{\mathrm{aug}}$ containing approximately equal numbers of positive and negative instances.
4.
$D_{\mathrm{aug}}$ is further partitioned into a training subset $D_{t}$ (90%) and a validation subset $D_{v}$ (10%), again using stratified sampling. Special care is taken to ensure enhancer–promoter pair indices are non-overlapping across splits to avoid data leakage.
5. The model is trained on $D_{t}$, validated on $D_{v}$, and finally evaluated on the original imbalanced test set $D_{\mathrm{test}}$—in line with prior EPI prediction benchmarks.

A summary of label distributions in $D_{\mathrm{aug}}$ and $D_{\mathrm{test}}$ for each cell line is provided in Table 1. To enrich the model with regulatory context, we also incorporate cell-type-specific genomic features. Specifically, we use five epigenomic signals—CTCF, DNase, H3K27ac, H3K4me1, and H3K4me3—which are known to reflect chromatin accessibility and histone modifications associated with regulatory activity. These signals are extracted from public repositories and transformed into fixed-length numerical vectors via a binning and normalization procedure inspired by Chen *et al*. [4].

### Feature extraction

**Figure 1.**
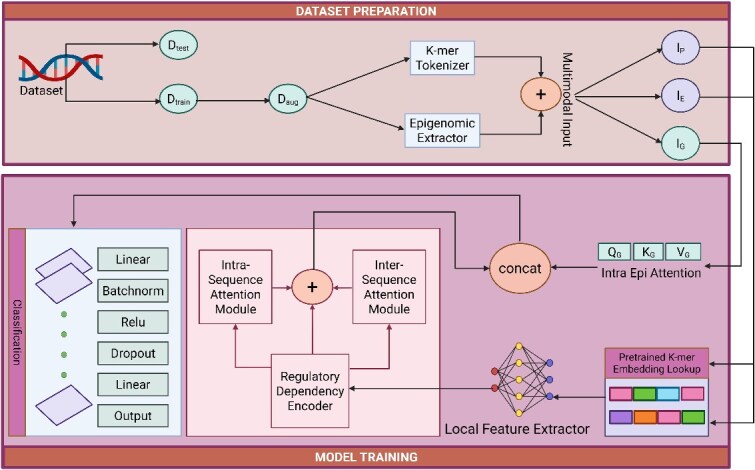
Overview of the EPINTLM architecture for EPI prediction.

This model incorporates two primary types of inputs: **sequence features** and **genomic features**, as illustrated in Fig. 1. Each modality is processed separately and later fused within the model architecture.

Sequence features. Enhancer and promoter sequences are first tokenized into overlapping 6-mers. Each unique 6-mer ($4^{6} = 4096$) is passed through a pretrained Nucleotide Transformer [9] in evaluation mode, and the final hidden states are mean-pooled to generate contextualized embeddings that capture local motifs and long-range dependencies. These embeddings form a static lookup table used to initialize the model’s embedding layer. During training, this layer is fine-tuned (requires_grad=True) to adapt the representations to EPIs prediction.

Genomic features. To incorporate regulatory context, we include five epigenomic signals known to reflect chromatin accessibility and regulatory activity: CTCF, DNase, H3K27ac, H3K4me1, and H3K4me3. Following the previous strategy [4], these signals are extracted from BED and BigWig files using fixed genomic windows around the enhancer and promoter regions. The extracted values are binned and normalized to produce fixed-length features, which are concatenated with sequence embeddings during multimodal feature fusion.

### Model architecture

We present EPINTLM (Enhancer–Promoter Interaction Nucleotide Transformer Large Model), a novel architectural framework for EPI prediction. The comprehensive model architecture is illustrated in Fig. 1.

Our architectural design is inspired by the EPIVAN and EPIPDLF methodologies [2, 8] that utilize embedding matrices derived from pretrained models. However, this study extends the methodology by incorporating the Nucleotide Transformer pretrained model as a substitute for DNA2vec [7] or DNABERT [11], enabling feature extraction across all 6-mers and applying mean pooling to the final layer to obtain contextualized embeddings for each 6-mer. The architecture is further enhanced through the integration of residual connections and cross-attention mechanisms. Additionally, we propose an alternative data preprocessing pipeline that significantly improves training efficiency while reducing computational complexity.

#### Efficient data preprocessing pipeline

To improve training efficiency and reduce computational overhead, we designed a streamlined data preprocessing pipeline for large-scale EPI datasets. Instead of storing sequence and genomic features separately, all tokenized enhancer and promoter sequences, genomic features, and labels are aggregated into a single SeqGenDataset. Sequence tokenization is parallelized for speed, and all processed data are saved as contiguous tensors in one .pt file. This design enables fast, index-based access during training and avoids repeated disk reads or data merging (see Algorithm 1).



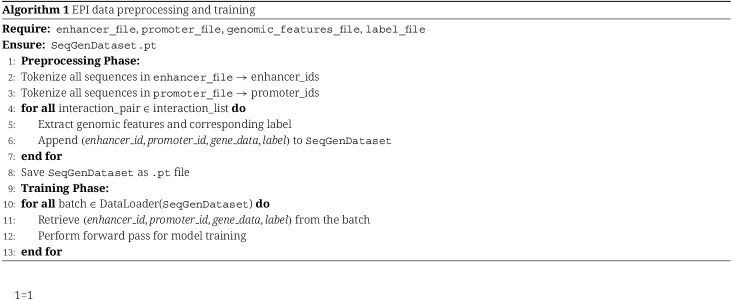



#### Embedding layer

To represent DNA sequences in a high-dimensional, information-rich space, both enhancer and promoter sequences are first tokenized into overlapping 6-mers. For a batch size of 256, the enhancer and promoter data are represented as $\mathbf{I}_{E} \in \mathbb{N}^{256 \times 2995}$ and $\mathbf{I}_{P} \in \mathbb{N}^{256 \times 1995}$, where each integer denotes a unique 6-mer.

Pretrained K-mer embedding lookup: We construct an embedding matrix $\mathbf{E} \in \mathbb{R}^{4097 \times 1280}$ by applying the Nucleotide Transformer to all 4096 possible 6-mers, plus one special token. For each batch, enhancer and promoter sequences are represented as integer-indexed tensors $\mathbf{I}_{E} \in \mathbb{N}^{256 \times 2995}$ and $\mathbf{I}_{P} \in \mathbb{N}^{256 \times 1995}$, and embedded via lookup: 


(1) \begin{align*} \mathbf{X}_{E} &= \mathbf{E}[\mathbf{I}_{E}] \in \mathbb{R}^{256 \times 2995 \times 1280} \end{align*}

(2) \begin{align*} \mathbf{X}_{P} &= \mathbf{E}[\mathbf{I}_{P}] \in \mathbb{R}^{256 \times 1995 \times 1280} \end{align*}

Fine-tuning strategy: To combine generalization and task-specific adaptation, the embedding matrix is frozen for the first two epochs. From the third epoch onward, we unfreeze the matrix, allowing it to be fine-tuned during training.

#### Local feature extractor

Following the embedding step, we use a 1D convolutional neural network (CNN) [12] to detect important local nucleotide motifs in enhancer and promoter sequences. CNNs are highly effective in recognizing biologically relevant motifs by capturing local patterns within the DNA sequences, thus converting raw nucleotide sequences into informative local features [13]. The model identifies crucial regions linked to regulatory activity by capturing these features, which are then used as important inputs for the following modules. For each embedded sequence tensor, denoted as $\mathbf{X} \in \mathbb{R}^{B \times L \times d}$ (where $B$ is the batch size, $L$ is the sequence length, and $d$ is the embedding dimension), we first permute the dimensions to match CNN input requirements: $\mathbf{X}_{\mathrm{cnn}} \in \mathbb{R}^{B \times d \times L}$. A one-dimensional convolutional operation is then applied with a kernel size of 40 and 64 output channels, followed by a ReLU activation function, max pooling with a kernel size of 20, batch normalization, and dropout for regularization. This process is performed independently for both enhancer and promoter inputs, resulting in: $\mathbf{C}_{E} \in \mathbb{R}^{B \times 64 \times L_{E}^{\prime}},\quad \mathbf{C}_{P} \in \mathbb{R}^{B \times 64 \times L_{P}^{\prime}}$ where $L_{E}^{\prime}$ and $L_{P}^{\prime}$ are the reduced sequence lengths after convolution and pooling. Specifically, given the input lengths of 2995 for enhancers and 1995 for promoters, the convolution (kernel size 40, stride 1) followed by max pooling (kernel size 20, stride 20) reduces them to $L_{E}^{\prime} = 147$ and $L_{P}^{\prime} = 97$, respectively.

#### Regulatory dependency encoder

We employ a two-layer bidirectional GRU [14] to capture sequential dependencies in enhancer and promoter sequences. The convolutional outputs $\mathbf{C}_{E} \in \mathbb{R}^{B \times 64 \times L_{E}^{\prime}}$ and $\mathbf{C}_{P} \in \mathbb{R}^{B \times 64 \times L_{P}^{\prime}}$ (147 and 97 positions, respectively) are permuted to $[L_{E}^{\prime}, B, 64]$ and $[L_{P}^{\prime}, B, 64]$ so that each time step corresponds to a sequence position represented by a 64-dimensional vector. The bidirectional GRU processes sequences in both forward and backward directions, yielding hidden states that integrate past and future context. With a hidden size of 32 per direction, this layer outputs $\mathbf{H}_{E} \in \mathbb{R}^{L_{E}^{\prime} \times B \times 64}$ and $\mathbf{H}_{P} \in \mathbb{R}^{L_{P}^{\prime} \times B \times 64}$. After that, we apply two attention branches working concurrently on the GRU-encoded representations: a self-attention branch for intra-sequence refinement and a cross-attention branch for inter-sequence interaction modeling.

#### Intra-sequence attention module and intra-EPI attention module

To effectively model the internal dependencies within each input type, we introduce two attention-based modules: Intra-Sequence Attention Module and Intra-Epigenomic Attention Module. In this stage, we apply multi-head self-attention [5] independently to the enhancer, promoter, and genomic inputs. This allows the model to focus on contextually important nucleotide positions within the sequences and to capture interactions among different regulatory signals within the epigenomic features. These attention mechanisms refine each representation before they are passed to subsequent components of the model. The inputs to the Intra-Sequence and Intra-Epigenomic Attention Modules include the enhancer representation $\mathbf{H}_{E} \in \mathbb{R}^{L^{\prime}_{E} \times B \times d}$, the promoter representation $\mathbf{H}_{P} \in \mathbb{R}^{L^{\prime}_{P} \times B \times d}$, and the genomic feature representation $\mathbf{G} \in \mathbb{R}^{1 \times B \times d}$, where $L^{\prime}_{E}$ and $L^{\prime}_{P}$ denote the pooled sequence lengths of the enhancer and promoter, respectively, $B$ is the batch size, and $d = 64$ is the feature dimension. We apply multi-head self-attention separately to each of the three feature types: 


(3) \begin{align*} \mathbf{S}_{E} &= \mathrm{SelfAttn}(\mathbf{H}_{E}) \in \mathbb{R}^{L_{E}^{\prime} \times B \times d} \end{align*}

(4) \begin{align*} \mathbf{S}_{P} &= \mathrm{SelfAttn}(\mathbf{H}_{P}) \in \mathbb{R}^{L_{P}^{\prime} \times B \times d} \end{align*}

(5) \begin{align*} \mathbf{S}_{G} &= \mathrm{SelfAttn}(\mathbf{G}) \in \mathbb{R}^{1 \times B \times d} \end{align*}

Here, for each input type—enhancer, promoter, and epigenomic signal representations—self-attention computes the output at each position as a weighted sum over all positions within the same input. While enhancer and promoter inputs contain multiple positions and benefit from contextual modeling, the genomic input consists of a single vector; in this case, the attention layer effectively acts as a multi-path linear transformation. Specifically, for the enhancer: 


(6) \begin{align*} \mathbf{q}_{i}^{(h)} &= \mathbf{h}_{E,i} \mathbf{W}_{Q}^{(h)},\ \mathbf{k}_{j}^{(h)} = \mathbf{h}_{E,j} \mathbf{W}_{K}^{(h)},\ \mathbf{v}_{j}^{(h)} = \mathbf{h}_{E,j} \mathbf{W}_{V}^{(h)}\end{align*}

where $h$ indexes attention heads, and $\mathbf{W}_{Q}^{(h)}, \mathbf{W}_{K}^{(h)}, \mathbf{W}_{V}^{(h)} \in \mathbb{R}^{d \times d_{a}}$. All head outputs are concatenated and projected: 


(7) \begin{align*} \mathbf{S}_{E,i} &= \mathrm{Concat}(\mathbf{o}_{i}^{(1)},..., \mathbf{o}_{i}^{(H)}) \mathbf{W}_{O}\end{align*}

where $H$ is the number of heads, and $\mathbf{W}_{O} \in \mathbb{R}^{H d_{a} \times d}$. Similarly, for promoter and genomic data.

### Inter-sequence attention module

After the bidirectional GRU block, we also introduce the Inter-Sequence Attention Module to capture interactions between enhancer and promoter representations. In particular, we use multi-head cross-attention [5] in both directions, enabling promoter features to be attended to by enhancer features and vice versa. As a result, the model can acquire the cross-regulatory dependencies necessary for precise prediction of EPIs. Given the representations $\mathbf{H}_{E}$ and $\mathbf{H}_{P}$ obtained from the *Regulatory Dependency Encoder*, the cross-attended outputs are computed as: 


(8) \begin{align*} \mathbf{C}_{E} &= \mathrm{CrossAttn}(\mathbf{H}_{E}, \mathbf{H}_{P}) \in \mathbb{R}^{L_{E}^{\prime} \times B \times d} \end{align*}

(9) \begin{align*} \mathbf{C}_{P} &= \mathrm{CrossAttn}(\mathbf{H}_{P}, \mathbf{H}_{E}) \in \mathbb{R}^{L_{P}^{\prime} \times B \times d} \end{align*}

For cross-attention from enhancer to promoter, queries are $\mathbf{H}_{E}$, keys and values are $\mathbf{H}_{P}$. For each head $h$ and for each enhancer position $i$: 


(10) \begin{align*} \mathbf{q}_{i}^{(h)} &= \mathbf{H}_{E,i} \mathbf{W}_{Q}^{(h)}, \mathbf{k}_{j}^{(h)} = \mathbf{H}_{P,j} \mathbf{W}_{K}^{(h)}, \mathbf{v}_{j}^{(h)} = \mathbf{H}_{P,j} \mathbf{W}_{V}^{(h)}\end{align*}

The head outputs are concatenated and linearly projected as above. Cross-attention allows each enhancer position to selectively aggregate information from all promoter positions (and vice versa), thereby capturing inter-sequence dependencies.

Residual connections and output aggregation: After that, residual connections are used to aggregate the outputs of the GRU, self-attention, and cross-attention modules for both the enhancer and promoter branches. These connections facilitate stable training and allow the model to preserve and combine both local and contextual information learned at different levels of representation. 


(11) \begin{align*} \mathbf{H}_{E}^{*} &= \mathbf{H}_{E} + \mathbf{S}_{E} + \mathbf{C}_{E} \quad \in \mathbb{R}^{L_{E}^{\prime} \times B \times d} \end{align*}

(12) \begin{align*} \mathbf{H}_{P}^{*} &= \mathbf{H}_{P} + \mathbf{S}_{P} + \mathbf{C}_{P} \quad \in \mathbb{R}^{L_{P}^{\prime} \times B \times d} \end{align*}

(13) \begin{align*} \mathbf{G}^{*} &= \mathbf{S}_{G} \quad \in \mathbb{R}^{1 \times B \times d} \end{align*}

These are then used in downstream feature fusion and classification modules.

#### Feature fusion and classification

After obtaining the enriched representations from the previous attention modules, we integrate the features from the enhancer, promoter, and genomic data to perform the final prediction of EPI.

Feature fusion: We concatenate the contextually enhanced representations along the sequence dimension: 


(14) \begin{align*}& \mathbf{F} = \mathrm{Concat}(\mathbf{H}_{E}^{*}, \mathbf{H}_{P}^{*}, \mathbf{G}^{*}) \in \mathbb{R}^{(L_{E}^{\prime} + L_{P}^{\prime} + 1) \times B \times d}\end{align*}

The concatenated tensor is then reshaped for classification module, typically as $\mathbf{F}^{\prime} \in \mathbb{R}^{B \times ((L_{E}^{\prime} + L_{P}^{\prime} + 1) d)}$.

Loss function: The model is trained end-to-end using the binary cross-entropy loss between the predicted probabilities and the ground truth interaction labels. Formally, for a batch of $B$ samples, let $\hat{y}_{i} \in [0,1]$ be the predicted probability and $y_{i} \in \{0,1\}$ the true label for the $i$th sample. The loss function is: 


(15) \begin{align*}& \mathcal{L}_{\mathrm{BCE}} = -\frac{1}{B} \sum_{i=1}^{B} \left[ y_{i} \log(\hat{y}_{i}) + (1 - y_{i}) \log(1 - \hat{y}_{i}) \right]\end{align*}

where $\hat{y}_{i} = \mathrm{MLP}(\mathbf{F}_{i}^{\prime})$ is the model output for sample $i$, $\mathrm{B} = 256$. All model parameters are optimized by minimizing $\mathcal{L}_{\mathrm{BCE}}$ over the training set.

## Experimental results

### Experimental setup

We evaluated model performance using AUROC and AUPR [15]. The model was trained with a batch size of 256 using Adam (weight decay = 0.001) and MultiStepLR. The learning rate was $1 \times 10^{-3}$, reduced to $1 \times 10^{-4}$ after unfreezing embeddings. Training required at most 1.5 h per cell line.

### Performance comparison across cell lines

We evaluated EPINTLM against five state-of-the-art predictors (EPIPDLF, EPIANN, SIMCNN, PEP-WORD, and SPEID) using six human cell lines from the TargetFinder dataset. All models were trained and tested on the same datasets for each cell line. The training and testing procedures were conducted for each comparative method according to the descriptions provided in their original references. As shown in Table 2 and Supplementary Fig. S1, EPINTLM achieved the highest AUROC values in four cell lines (HUVEC: 0.935, HeLa: 0.970, K562: 0.947, andGM12878: 0.949). EPINTLM leveraged transformer architecture to effectively model the long-range dependencies critical for EPI prediction [4]. Unlike CNN-based approaches that struggle with capturing distant relationships, our approach that integrated self-attention and cross-attention excels at learning the complex interactions between enhancers and promoters across large genomic distances [16, 17].

**Table 2:** Benchmark performance comparison of EPINTLM and existing methods across six human cell lines

**Model**	**GM12878**		**HELA**		**HUVEC**		**IMR90**		**K562**		**NHEK**	
	AUC	AUPR	AUC	AUPR	AUC	AUPR	AUC	AUPR	AUC	AUPR	AUC	AUPR
EPIANN	0.919	0.723	0.924	0.702	0.918	0.616	0.945	0.770	0.943	0.673	0.959	0.861
SIMCNN	0.941	0.706	0.949	0.737	0.933	0.640	**0.951**	0.737	0.943	0.679	0.962	0.882
PEP-WORD	0.842	**0.807**	0.843	0.803	0.845	**0.760**	0.898	**0.868**	0.883	**0.836**	0.917	0.880
SPEID	0.916	0.773	0.923	0.797	0.904	0.523	0.915	0.732	0.922	0.771	0.950	0.852
EPIPDLF	0.939	0.788	0.964	0.849	**0.935**	0.730	0.936	0.779	0.943	0.755	**0.993**	**0.925**
**EPINTLM (Ours)**	**0.949**	0.779	**0.970**	**0.865**	**0.935**	0.741	0.909	0.727	**0.947**	0.771	0.985	0.907

Bold indicates the best-performing value for each metric within a given cell line.

Notably, EPINTLM achieved strong AUROC and AUPR performance on the HeLa cell line (Table 2 and Supplementary Fig. S1), improving AUC by 4.98%, 2.21%, 15.07%, 5.09%, and 0.62% over EPIANN, SIMCNN, PEP-WORD, SPEID, and EPIPDLF, respectively. However, HeLa cells are characterized by extensive oncogenic transformation and highly aberrant regulatory programs, and therefore should not be regarded as representative of normal enhancer–promoter regulation [18, 19]. In this study, performance on HeLa is interpreted as an architectural stress test under transcriptionally dense and highly perturbed conditions rather than as evidence of biological generalizability. In such settings, enhancer and promoter regions often contain dense regulatory signals, increasing the risk of information degradation across deep layers. The incorporation of residual connections helps stabilize learning under these conditions, while explicit cross-attention enables reciprocal information exchange between enhancer and promoter representations within the benchmark evaluation framework.

Regarding the AUPR metric presented in Table 2, EPINTLM achieved the highest score on the HeLa cell line, improving AUPR by 23.2%, 17.4%, 7.7%, 8.5%, and 1.9% over EPIANN, SIMCNN, PEP-WORD, SPEID, and EPIPDLF, respectively. On the HUVEC and K562 cell lines, EPINTLM marginally improved AUPR performance over EPIPDLF, with relative improvements of 1.5% and 2.1%. Although PEP-WORD attained the highest AUPR on four out of six cell lines, it recorded among the lowest AUC values across most cell lines. This observation suggests a trade-off in PEP-WORD between overall class discrimination and positive class classification, indicating that the model tends to focus more on identifying positive samples rather than distinguishing between the two classes. Despite this trade-off, PEP-WORD’s architecture may still explain its improved AUPR performance. Using two main modules (PEP-Motif and PEP-Word), it extracts sequence-based features and concatenates them as input for a Gradient Tree Boosting classifier [20] that probably improves positive class recognition. In particular, rather than using convolution-based feature learning as in EPINTLM, PEP-Motif computes the normalized occurrence frequencies of known TFBS motifs in enhancer and promoter sequences. In certain cell lines, EPINTLM’s AUPR is still constrained despite its strong AUC performance. To further improve AUPR without sacrificing AUC performance, future research may incorporate motif-occurrence frequency features and explore ensemble learning strategies (such as combining deep learning with tree-boosting models).

If not considering PEP-WORD, EPINTLM achieved the highest AUPR on the HELA, HUVEC, and K562 cell line while maintaining consistently high AUROC values (Table 2). This balanced performance indicates that EPINTLM can both accurately identify positive interactions and effectively distinguish between interacting and non-interacting pairs, which indicates balanced discrimination behavior under the current benchmark evaluation settings [16].

**Figure 2.**
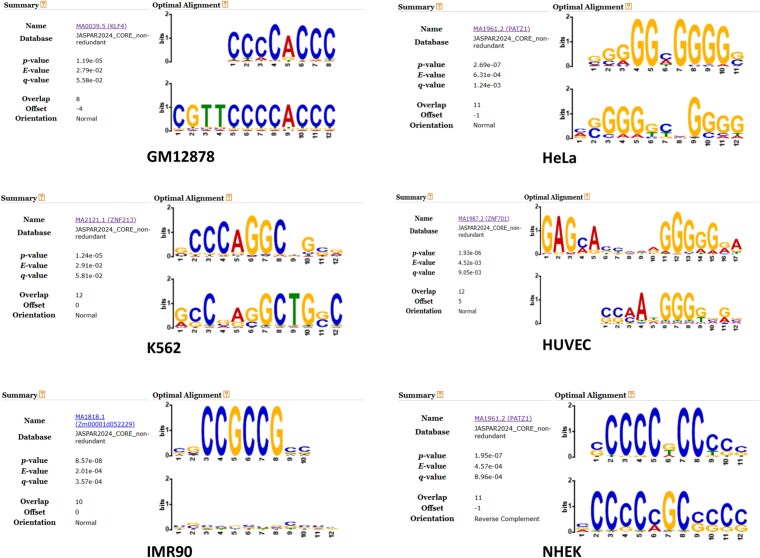
Sequence motifs highlighted by cross-attention analysis across six human cell lines.

IMR90 exhibits lower performance compared with the other cell lines that may be attributed to its smaller number of positive interactions (1254 positive and 25 000 negative cases) and weaker motif conservation. We further investigate this in the interpretability analysis (Fig. 2). This observation highlights the sensitivity of sequence-based EPI models to dataset-specific characteristics within the benchmark, rather than providing direct evidence of cell-type-specific regulatory mechanisms [2]. The reduced performance on IMR90 underscores the limitations of current benchmark datasets (Table 2) in representing diverse regulatory architectures and further emphasizes the need for richer contextual information and larger-scale resources in future evaluations [6, 16].

### Ablation study

We perform an ablation study by removing essential modules from the complete model for EPI prediction to verify the effectiveness of our suggested architecture components. Specifically, we compare three variants: (i) the complete model with all components intact, (ii) a reduced model without both residual connections and cross-attention modules, and (iii) a model that removes only the residual connections while retaining cross-attention. Table 3 reports the AUROC performance of the full model and its two ablated variants across six cell lines. The full model consistently outperforms both alternatives, with particularly notable improvements on most cell lines, except for HUVEC.

**Table 3:** AUROC performance of EPINTLM and ablated variants across six human cell lines

**Model**	**GM12878**	**HELA**	**HUVEC**	**IMR90**	**K562**	**NHEK**
Ours w/o Residual and Cross-Attn	0.943 $\pm $ 0.005	0.969 $\pm $ 0.008	0.928 $\pm $ 0.012	0.907 $\pm $ 0.006	0.936 $\pm $ 0.015	0.982 $\pm $ 0.004
Ours w/o Residual	0.939 $\pm $ 0.006	0.968 $\pm $ 0.005	**0.938 $\pm $ 0.006**	0.908 $\pm $ 0.008	**0.947 $\pm $ 0.003**	0.984 $\pm $ 0.002
EPINTLM (Ours)	**0.949 $\pm $ 0.002**	**0.970 $\pm $ 0.004**	$0.935 \pm 0.000$	**0.909 $\pm $ 0.003**	**0.947 $\pm $ 0.001**	**0.985 $\pm $ 0.004**

Values are reported as mean $\pm $ standard deviation across runs. Bold indicates the best performance for each cell line.

In terms of AUPR (Table 4), EPINTLM surpasses the two ablated variants on the HeLa cell line. On the HUVEC, IMR90, K562, and NHEK cell lines, the ours w/o Residual variant outperforms the other two, suggesting that the cross-attention mechanism (Inter-Sequence Attention Module) effectively enhances the model’s ability to identify positive cases. In contrast, residual connections appear to contribute less to improving AUPR than cross-attention.

**Table 4:** AUPR performance of EPINTLM and ablated variants across six human cell lines

**Model**	**GM12878**	**HELA**	**HUVEC**	**IMR90**	**K562**	**NHEK**
Ours w/o Residual and Cross-Attn	**0.787 $\pm $ 0.005**	0.861 $\pm $ 0.015	0.700 $\pm $ 0.027	0.724 $\pm $ 0.012	0.751 $\pm $ 0.040	0.910 $\pm $ 0.007
Ours w/o Residual	0.780 $\pm $ 0.012	0.863 $\pm $ 0.010	**0.748 $\pm $ 0.008**	**0.733 $\pm $ 0.008**	**0.772 $\pm $ 0.019**	**0.911 $\pm $ 0.008**
EPINTLM (Ours)	$0.779 \pm 0.001$	**0.865 $\pm $ 0.011**	$0.741 \pm 0.006$	$0.727 \pm 0.007$	$0.771 \pm 0.007$	$0.907 \pm 0.009$

Values are reported as mean $\pm $ standard deviation across runs. Bold indicates the best performance for each cell line.

Removing both modules reduces the model’s ability to tell the difference between classes (e.g. a 1.2% drop in AUROC on K562). It makes it much harder to find positive cases (e.g. a 5.5% drop in AUPR on HUVEC). These comparisons show how vital cross-attention and residual connections are to our overall architecture.

### Biologically meaningful motifs learned by EPINTLM

To enhance the interpretability of the model, we extracted sequence motifs learned by EPINTLM based on cross-attention from enhancers to promoters (Fig. 2 and Supplementary Fig. S2). Specifically, we analyzed the cross-attention weights from enhancer to promoter sequences for positive samples. We then extracted 12 bp subsequences with the highest scores, corresponding to promoter regions most strongly attended to by enhancers, and compared them against the JASPAR 2024 CORE database [21] using TOMTOM [22]. The learned motifs showed strong similarity to known transcription factor binding motifs, with consistently low P-values, E-values, and Q-values, as illustrated in Fig. 2. These results suggest that the model is capable of identifying biologically meaningful sequence motifs and that enhancers may focus their attention on important regions within promoters.

As shown in Table 2, EPINTLM achieves competitive AUC and AUPR scores on most cell lines, but drops sharply on IMR90. From the cross-attention weights in the enhancer-to-promoter direction, the model extracted only one motif for IMR90, while other cell lines yielded >10. The IMR90 motif is also weak, with nucleotide heights mostly <0.3 bits in Fig. 2, indicating low conservation and poor information content. This suggests that enhancer-attended promoter motifs are not well captured in this dataset. In future work, we may address this issue by incorporating epigenomic signals, domain-specific pretraining, or transfer learning.

## Discussion and Conclusion

In this study, we presented EPINTLM, a deep learning framework for EPI prediction that integrates DNA sequence representations with genomic features. The model combines pretrained k-mer embeddings derived from the Nucleotide Transformer with explicit modeling of inter-sequence dependencies through cross-attention and residual aggregation. When evaluated on a widely used benchmark across six human cell lines, EPINTLM achieved performance, i.e. competitive with, and in some cases modestly improved over, existing state-of-the-art methods. Ablation experiments further demonstrated that explicit cross-attention and residual connections contribute to stable learning and balanced AUROC–AUPR behavior. In addition, the proposed unified preprocessing pipeline improves computational efficiency and reproducibility, while post hoc motif analysis provides limited but interpretable insights into the sequence patterns leveraged by the model.

At the same time, several important limitations must be acknowledged. First, predictive performance varies across cell types. In particular, EPINTLM underperforms on IMR90, which is characterized by fewer positive interactions and weaker motif conservation. This observation suggests that cell-type-specific regulatory architectures and data sparsity remain challenging for sequence-centric EPI models. Addressing these issues may require incorporating richer regulatory context, such as transcription factor binding profiles, chromatin accessibility dynamics, or cell-type-aware adaptation strategies, rather than relying on sequence information alone.

More fundamentally, the scope of the conclusions drawn in this work is constrained by the benchmark dataset itself. The TargetFinder benchmark contains a relatively small number of experimentally supported positive interactions and represents only a tiny fraction of the potential enhancer–promoter pairs in the human genome. Moreover, enhancer–promoter regulation is inherently many-to-many, highly context-dependent, and condition-specific, properties that are not fully captured by current benchmark constructions. Consequently, EPINTLM should not be interpreted as a model of genome-wide regulatory wiring or as a solution to real-world EPI discovery. Instead, the present study should be viewed as a controlled methodological investigation, designed to assess architectural choices—particularly explicit bidirectional cross-attention—under standardized evaluation settings that are consistent with prior work in the field.

Looking forward, improving robustness across diverse cellular contexts and scaling evaluation beyond curated benchmark datasets remain critical challenges. Future work will explore extending the framework to larger and more heterogeneous resources, including ENCODE-derived and ligation-based datasets such as PLAC-seq, while carefully accounting for their increased biological complexity and labeling uncertainty. In parallel, alternative foundation models and multimodal architectures that jointly encode sequence, chromatin state, and regulatory topology may provide richer representations of enhancer–promoter relationships.

In conclusion, EPINTLM represents a methodological step toward more explicit modeling of enhancer–promoter dependencies within current benchmark constraints. While substantial challenges remain in translating such models to genome-scale regulatory inference, the results highlight the potential value of cross-attention-based designs for paired regulatory sequence modeling and provide a foundation for future exploration as data resources and evaluation paradigms continue to evolve.

**Key Points**

- EPINTLM is a deep learning framework for enhancer–promoter interaction prediction evaluated under standardized benchmark settings.
- The model uses bidirectional cross-attention and residual aggregation to explicitly model enhancer–promoter dependencies.
- Pretrained k-mer embeddings from a DNA foundation model improve sequence representation learning.
- Benchmark experiments across six human cell lines show competitive AUROC and AUPR performance.
- The study provides methodological insights while acknowledging the limitations of current benchmark datasets for genome-wide inference.

## Supplementary Material

Bioinformatic_Supp_final_bbag064

## Data Availability

Code and data are publicly available at https://github.com/langiocn/EPINTLM.
