# Supplementary material for: EPINTLM: enhancer–promoter prediction with pretrained k-mer embeddings and residual cross-attention
Source: Brief Bioinform. 2026 Feb 16;27(1):bbag064. doi: 10.1093/bib/bbag064 (PMC12908682; doi:10.1093/bib/bbag064)
Supplement: Bioinformatic_Supp_final_bbag064 [file bioinformatic_supp_final_bbag064.pdf]

Supplementary Material for: EPINTLM: Enhancer–Promoter Prediction with Pretrained k-mer Embeddings and Residual Cross-Attention

1. Additional Experiments

For a more detailed assessment of performance, we visualize the ROC and PR curves for each cell line in Figure S1. We can observe that NHEK achieves the highest AUC and AUPR scores, whereas IMR90 records the lowest overall performance. Although IMR90 has the smallest dataset, which may partially explain its weaker results, NHEK's superior performance cannot be attributed to data quantity alone. A possible reason is that the combination of 6-mer sequence features and epigenomic signals may be more informative and better captured by EPINTLM in this cell line.

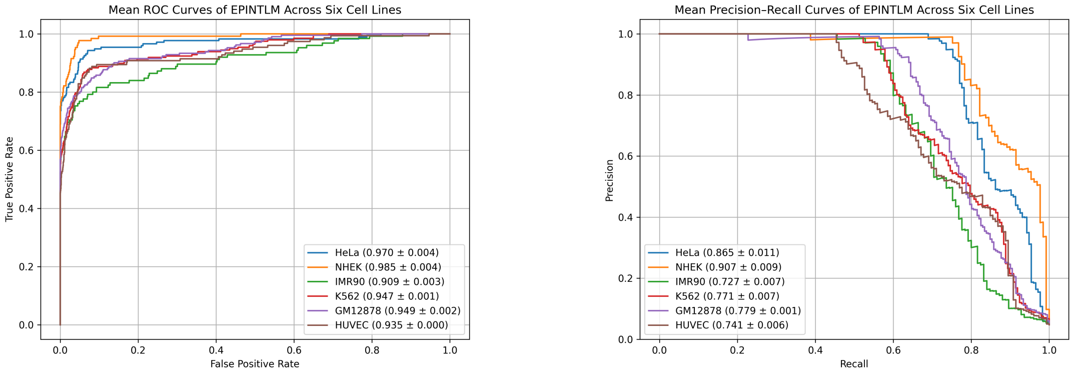

(A) ROC curves across six cell lines (B) PR curves across six cell lines

Figure S1: EPINTLM – Performance of our model on six cell lines.

EPINTLM's performance advantage stems not only from raw metrics but also from architectural innovations. Unlike prior CNN-based models such as SPEID Singh et al. (2019), EPINTLM uses cross-attention to directly model enhancer–promoter dependencies across long genomic distances, a critical feature in chromatin context modeling Vaswani et al. (2017). Residual connections, though less emphasized in some benchmarks, offer crucial signal stability across depth, particularly in transcriptionally dense cell types like HeLa.

© 2025 .

2. Extended Explanation

We analyzed the cross-attention weights from enhancer to promoter sequences for positive samples. Averaging across heads yielded a promoter saliency map, highlighting subsequences that were most strongly influenced by enhancer regions, as shown in Figure S2

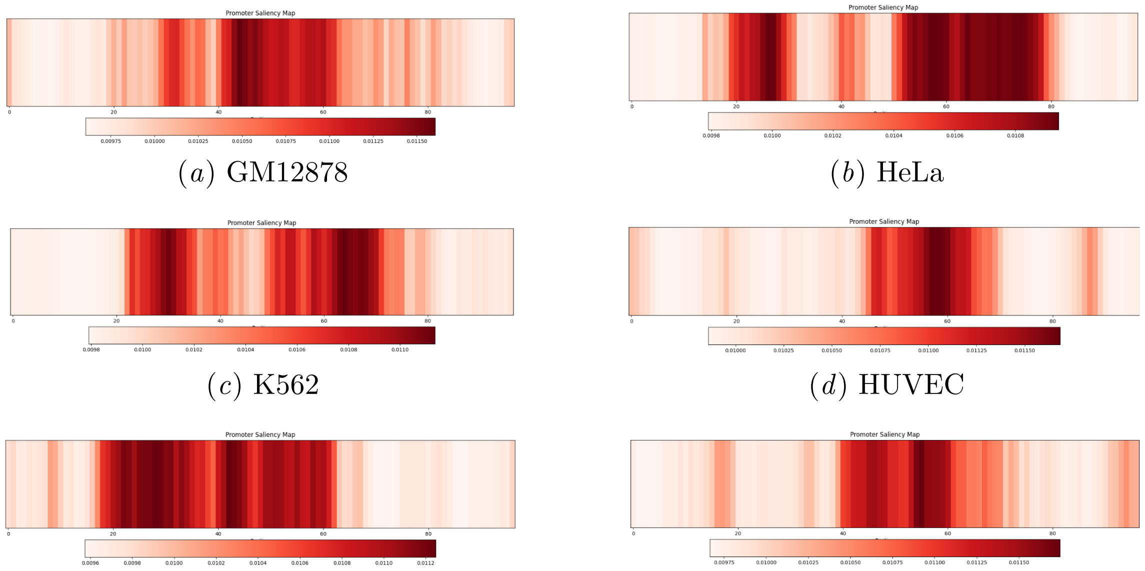

(a) GM12878 (b) HeLa (c) K562 (d) HUVEC (e) IMR90 (f) NHEK

Figure S2: Saliency map of promoter regions derived from enhancer-to-promoter cross-attention.

References

Shashank Singh, Yang Yang, Barnabás Póczos, and Jian Ma. Predicting enhancer-promoter interaction from genomic sequence with deep neural networks. *Quantitative Biology*, 7 (2):122–137, 2019.

Ashish Vaswani, Noam Shazeer, Niki Parmar, Jakob Uszkoreit, Llion Jones, Aidan N Gomez, Łukasz Kaiser, and Illia Polosukhin. Attention is all you need. *Advances in Neural Information Processing Systems*, 30, 2017.
